# Spinopelvic Fixation Supplemented With Gullwing Plate for Multiplanar Sacral Fracture With Spinopelvic Dissociation: A Case Series With Short Term Follow Up

**DOI:** 10.3389/fsurg.2019.00042

**Published:** 2019-07-19

**Authors:** Muhammad Azrin Mohd Asihin, Mohd Yazid Bajuri, Abdul Rauf Ahmad, Premganesh K. Ganaisan, Mohamad Fazir, Azizul Akram Salim

**Affiliations:** ^1^Department of Orthopedic, Hospital Tuanku Ja'afar, Seremban, Malaysia; ^2^Department of Orthopedics and Traumatology, Universiti Kebangsaan Malaysia Medical Centre, Hospital Canselor, Kuala Lumpur, Malaysia; ^3^Department of Orthopedic, Hospital Kuala Lumpur, Kuala Lumpur, Malaysia; ^4^Department of Orthopedic, Hospital Sultanah Nur Zahirah, Kuala Terengganu, Malaysia

**Keywords:** spinopelvic, dissociation, multiplanar, fixation, gullwing plate

## Abstract

We describe a series of three patients who sustained multiplanar sacral fracture with spinopelvic dissociation treated with bilateral triangle osteosynthesis supplemented with a gullwing plate. Multiplanar sacral fracture causes the sacrum to divide into two parts which in severe cases, fracture displacement results in neurological injury. Spinopelvic fixation supplemented with a gullwing plate surgical treatment is still a viable option with an acceptable outcome. The average waiting time prior to surgery is 3 weeks.

## Introduction

The sacrum serves as the base of the spinal column and contributes to the stability of the pelvic ring structure (1, 2). Compound fractures of the sacrum may lead to spinopelvic instability or dissociation (3, 4). Traumatic spinopelvic dissociation is characterized by two longitudinal fractures on both sides of the sacrum exiting at sacral alae bilaterally. The two are connected by a transverse fracture line across the sacrum. All of these fracture lines lie in a different sacral plane, hence the term multiplanar sacral fracture. It often leads to H-, U-, Y-, or λ-shaped fracture patterns. It causes the sacrum to divide into two parts, cephalad, and caudal/peripheral part (5, 6). The cephalad part consists of the central upper portion of the sacrum still attached to the lumbar spine, and the caudal part is the portion of the sacral alae still attached to the lower sacrum and anterior pelvic ring via the sacroiliac joint, bilaterally. In severe cases, fracture displacement results in neurological injury such as cauda equina syndrome.

We operated on three cases of an unstable multiplanar sacral fracture with spinopelvic dissociation within a month duration. All cases were a week apart from each other. Similar spinopelvic fixation construct was used in every patient, extending from lumbar L4 and L5 pedicles to iliac bones bilaterally, supplemented with a gullwing plate spanning both sacroiliacs joint.

## Case Series

The case series includes three patients with multiplanar sacral fractures with spinopelvic dissociation, each admitted, and treated in three different tertiary referral hospital in the country located in different states. All the patients had standard plain radiograph (pelvis anteroposterior, inlet, and outlet view; lumbosacral lateral view) and Computed Tomography (CT) scan with a 3D reconstruction of the pelvis.

One patient had a BMI of 50 (body weight 145 kg) at the time of the surgery. None of the patients had neurological deficit preoperatively. All cases were encountered within the same month and were operated one after another within a week apart and were operated on by the same surgeons. Average waiting time to surgery was 3 weeks (ranges between 2 and 4 weeks).

### CASE 1

The patient was an 18-year-old who sustained U-shaped sacral fracture resulting in spinopelvic dissociation following a motorcycle accident. He was initially managed with iliac crest pelvic external fixator while hemodynamically stabilized in the intensive care unit. He underwent spinopelvic surgical stabilization 3 weeks following the initial trauma.

**Figure 1:**
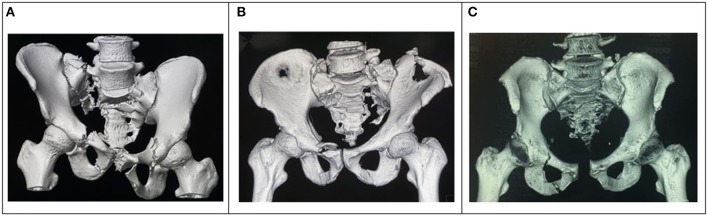
**(A)** Pelvis 3D reconstruction of an 18-year-old with unstable U-shaped multiplanar sacral fractures with spinopelvic dissociation. **(B)** Pelvic 3D reconstruction of the second patient with a U-shaped multiplanar sacral fracture with spinopelvic dissociation, and left iliac crest open fracture. **(C)** 3D reconstruction image of the pelvis depicting the injury pattern, a U-shaped multiplanar sacral fracture.

Based on the CT scan (Figure 1A), three fractures lines visible in the sacrum creating an unstable U-shaped sacral fracture with spinopelvic dissociation. One longitudinal fracture in Dennis zone 2 on the right side of the sacrum communicates with the second longitudinal fracture on the left side via a transverse fracture line through S1/S2. Posteriorly, the fracture pattern appears to spare the L5/S1 facet joint as both longitudinal fractures exit posteriorly just lateral to it. Anteriorly, there was bilateral superior and inferior pubic rami fracture; however, the pubic symphysis was not disrupted.

**Figure 2:**
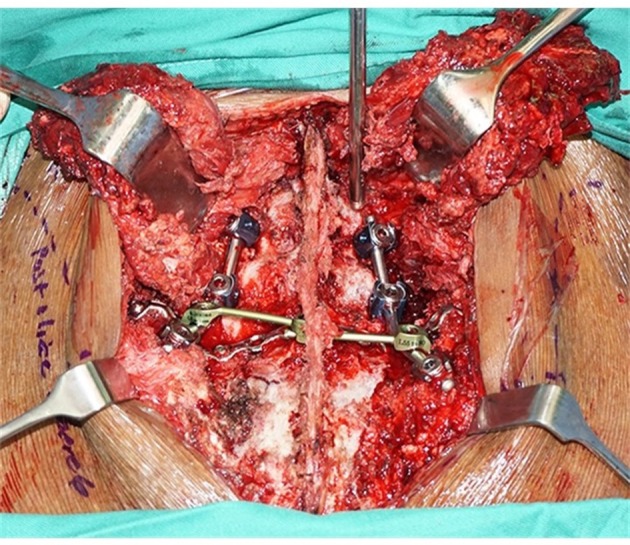
Final spinopelvic fixation constructs. Two pair of pedicle screws in L4 and L5 vertebrae, a pair of iliac screws, supplemented with a gull-wing plate (blue arrow).

The absence of L5/S1 facet fracture or dislocation in this first patient made the surgical fixation straightforward. Two pairs of polyaxial screws in L4 and L5 pedicles, and two long polyaxial screws inserted into both iliac wings. Two 5.5 mm stainless steel rod used to connect all the polyaxial pedicle screws. A gullwing plate was fashioned from a straight 3.5 mm reconstruction plate and implanted to supplement the final construct (Figure 2). The anterior pelvic ring disruption was managed with a supra-acetabular pelvic external fixator for 4 weeks.

### CASE 2

A 40-year-old gentleman who had a motor vehicle accident was admitted with an unstable U-shaped sacral fracture with an open fracture of the left iliac crest. He was haemodynamically unstable on admission, requiring resuscitation in intensive care unit, and initially managed with wound debridement, screw fixation of the left iliac crest and temporary stabilization of the pelvic ring with a pelvic external fixator.

The patient had a similar U-shaped fracture pattern to the sacrum with a transverse fracture occurring at the level of S3 (Figure 1B). Unlike the first patient, this patient, unfortunately, had both longitudinal fracture line exits posteriorly through the L5/S1 facet joint, resulting in a dislocation of L5/S1 facets bilaterally, causing L5/S1 instability. Also, the presence of the right transverse process fracture of L5 made it somewhat challenging to identify the point of entry of L5 pedicles screws bilaterally as the anatomy now disrupted—more time was spent in locating the point of insertion for pedicle screws of L5 segment.

He underwent similar surgical fixation 28 days following the initial trauma. It is of importance to point out that there was very minimal or absence of callus formation at the fracture site for this patient, despite the delay in surgical treatment. Similar findings were noted in the other two cases.

### CASE 3

**Figure 3:**
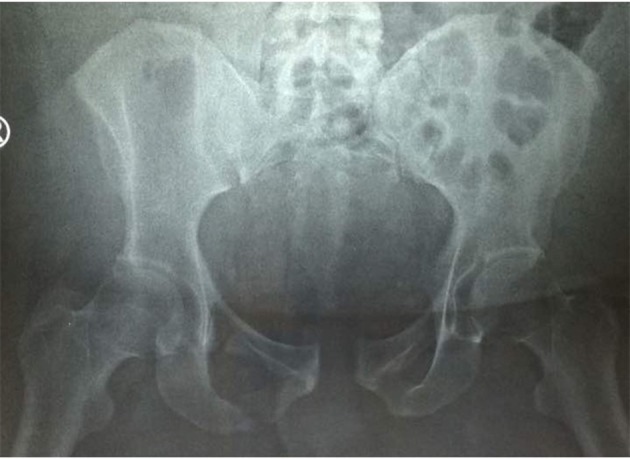
Pelvis AP radiograph of the patient with a BMI of 50 showing unclear findings of sacral bone pathology.

The third patient was a 44-year-old gentleman with a body weight of 150 kg and BMI of 50 (class 3 obesity). He was involved in a motor vehicle accident and presented with severe lower back pain, just above the tail bone. In the anterior ring, he suffered a fracture of the right superior and inferior pubic rami with pubic symphysis separation of <2 cm (Figure 3). Posteriorly, the right sacroiliac joint widened, however, it was uncertain if the sacrum was fractured based on plain radiograph.

The patient underwent a CT scan of the pelvis (Figure 1C) when the severe lower back pain persists despite adequate maximal analgesia prescribed. The CT scan which reveals an unstable lambda type sacrum fracture with spinopelvic dissociation. The unstable fracture pattern plays a significant role in the decision to proceed with surgical fixation for this morbidly obese patient, even though the sacrum fracture appears to be non-displaced.

**Figure 4:**
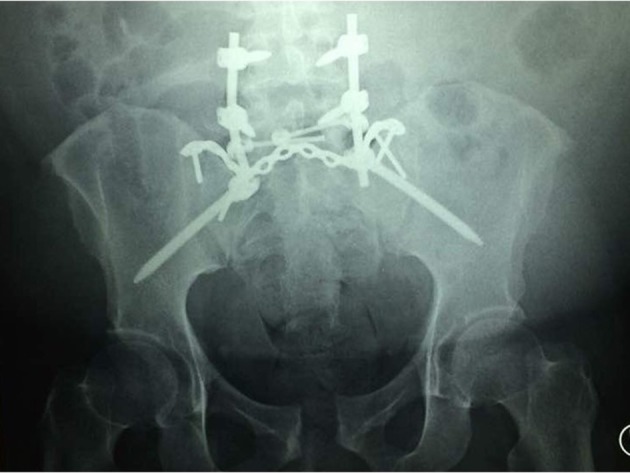
Spinopelvic fixation construct of the third patient. A similar construct was implanted in all 3 patients.

There is a concern of wound healing as this patient falls under class 3 obesity. However, a non-surgical approach is not favorable choice of treatment as risks of developing complication from immobilization is high due to his obesity. He had the same fixation construct as the other patients (Figure 4), which was done 2 weeks following injury. Anteriorly, the disrupted pubic symphysis treated conservatively as intraoperatively it was assessed and deemed stable without further displacement with stress test.

## Surgical Method

Preoperatively, all patients receive low molecular weight heparin (Enoxaparin 40 mg daily) to prevent venous thromboembolism and stopped prior to surgery. Surgery performed under general anesthesia in a prone position, and all patients received a single dose of IV cefuroxime 1.5 G antibiotic prophylaxis at induction. The knees and hip were supported and kept at a semi-flexed position between 15–30 degrees.

The surgical approach is through a straight midline incision between L4 spinous process down to S4 spine. Proximally, paralumbar muscles elevated subperiosteally to visualize entry point for insertion of two pair of 6.5 mm polyaxial pedicle screws into L4 and L5 pedicles. Distally, the lumbosacral musculature elevated as distal V-shaped flap bilaterally to reveal the posterior superior iliac spine (PSIS) and expose the sacral fracture. Using a bone rongeur, a recess created at the point of insertion of the iliac screws just medial to PSIS to prevent prominent screw head. Under fluoroscopy guidance, using a straight pedicle probe, a pre-drilling hole made directed toward the anterior inferior iliac spine (AIIS), passing ~2 cm above the greater sciatic notch, an area with dense cortical bone for better screw purchase. The screw hole made complete with a 4.5 drill bit before insertion of a 7.0 mm long polyaxial iliac screws bilaterally. The length of the iliac screw ranges between 90–110 mm.

The displaced sacral fracture then reduced with a combination of longitudinal traction, pushing, and pulling using the iliac screws and pedicle screws as lever. Once fracture reduction is satisfactory, a pair of 5.5 mm stainless steel rod prebent to recreate lordosis of the lower lumbar before securing them to all 6 polyaxial screws. A transverse cross-link between the 2 rods secured in between S1 and S2. Finally, a 3.5 mm reconstruction plate is then contoured to form a gullwing shape and implanted immediately posterior to the S1 segment or posterosuperior to the sacral alae to span both sacroiliac joints. At least one screw secured into each sacral ala and another into the PSIS bilaterally to secure the plate in place. A drain secured underneath paraspinal musculature bilaterally, followed by closure in layers. Postoperatively, VTE prophylaxis discontinued once the patient can tolerate limb physiotherapy and able to mobilize with a wheelchair. All three patients discharged within 1 week postoperatively.

A written informed consent was obtained from the participant for the publication of this case report. The case report has been approved by The Hospital Board from Hospital Tuanku Ja'afar, Seremban, Negeri Sembilan, Malaysia for ethical purposes.

## Results

All three patients had a follow up at 10 months following surgery. None had neurological deficit both preoperatively and postoperatively. Neurological assessment was performed using International Standards for Classification of Spinal Cord Injury (ISNCSCI) by American Spinal Injury Association (7). A functional assessment of the patients was performed using Majeed Pelvic Score (8). The first two scored 94 and 96 (excellent outcome). While the patient with BMI 50 scored 52 (fair outcome).

The first patient was a young patient with high motivation to return to an active lifestyle. At the time of assessment, he was ambulating without walking aid, able to squat down, and did not exhibit any neurological deficit despite having right sided sacral fracture in Denis zone 2.

The second patient had the most unstable fracture pattern among the three patients, as both L5/S1 facet joint fractured and dislocated. The instability between L5 and S1 segment was confirmed intraoperatively. However, the outcome in this patient was the most outstanding where he scores the highest, 96 points. On assessment at 10 months, he was able to walk without aid, squat without assistance, and was able to run. He did not display any neurological deficit on assessment. He was active and worked in a production factory.

The last patient, however, did not do well as compared to the other two patients, although his fracture was the least displaced. At the time of assessment, he was ambulating with a walking frame at home and uses a wheelchair for community ambulation. He persistently experiences mild to moderate lower back pain with an extended upright position, relieved with lying down. He scores 52 points with Majeed Pelvic Score, a fair outcome. None of our patients developed surgical site complication, reoperation, or neurological deficit postoperatively.

## Discussion

A non-operative approach was the preferred choice of treatment for this type of fractures in the past due to lack of reliable surgical technique and appropriate implants available at that time. However, this has changed over time with the advent of new techniques and the availability of new implants. Restoring the spinopelvic stability is the primary objective of surgical management of such fracture. Recent findings demonstrated that surgical treatment is superior in terms of short and long- term benefits in comparison to conservative non-operative treatment (3–6).

In 1999, Taguchi et al. reported 12 patients with displaced sacral fractures treated surgically. He described sacral fractures as having a longitudinal and transverse type and that bilateral vertical sacrum fractures were always associated with a transverse fracture (9). We now know that this is unstable H-type multiplanar sacral fracture and is associated with spinopelvic dissociation. He described fixation of the isolated vertical fractures of the sacrum using Iliosacral screw (ISS) and a plate extending from the sacrum to lower lumbar to treat the transverse sacral fracture.

Schildhauer et al. introduced triangular osteosynthesis for unstable sacral fracture. In a cadaveric study, he found that triangular osteosynthesis is far superior biomechanically in comparison to the traditional fixation with an iliosacral screw (ISS) alone in treating an isolated transforaminal sacral fracture (3). His construct consists of a pedicle screw in L5 spine connected to an ipsilateral iliac screw using a rod and supplemented with an ipsilateral iliosacral screw. The construct bypasses both fractured sacrum and disrupted sacroiliac joints. He proceeds to describe spinopelvic fixation using 2 pairs of pedicle screws in L4 and L5 spine and 2 points of iliac screw fixation bilaterally either with iliac screws alone or in combination with iliosacral screws (10).

Lumbopelvic fixation is the standard choice of treatment for cases of unstable multiplanar sacral fracture with spinopelvic dissociation (9, 11–13). Recently gaining popularity, lumbopelvic fixation construct being bilateral triangular osteosynthesis with lumbar pedicle screws (L4 and L5 vertebrae) and a pair of iliac screws connected with rods and commonly supplemented with an iliosacral screw (ISS) to maintain sacral fracture reduction (9, 11, 12, 14–16). However, the use of iliosacral screws is limited to non-comminuted and minimally displaced multiplanar sacral fractures (17, 18). The use of iliosacral screws for comminuted sacral fracture occurring in Denis Zone 2, may inadvertently results in compression of the fracture site and causing iatrogenic nerve injury.

Biomechanically, a triangular osteosynthesis is superior to iliosacral screw alone in fixation of unstable vertical transforaminal sacral fracture (3, 18). In one study, a gullwing plate was found to be biomechanically superior in comparison to a single iliosacral screw (19). A cadaveric biomechanical analysis of a gullwing plate to treat longitudinal fractures of the sacrum showed that the plate is biomechanically stable during postoperative loading and may enable patients to attain early mobility (20). Based on currently available literature, there is no known report of lumbopelvic fixation supplemented with a gullwing plate.

In our experience, a gullwing plate is a suitable replacement for iliosacral screw to supplement lumbopelvic fixation of unstable multiplanar sacral fracture with spinopelvic dissociation. Furthermore, the plate's screws direction is dynamic and can be adjusted according to fracture configuration. We used a 3.5 mm non-locking reconstruction plate bent and contoured to a gullwing shape to sit directly posterior on the S1 vertebra. The objective of contouring the plate is to have as much bone-implant contact between the S1 vertebra and gullwing plate.

In average, the waiting period for all our patients was 3 weeks with one of the patients operated 28 days following the trauma. Despite the long waiting period before surgery, the fracture site for all patients were mobile with minimal to no callus formation encountered during surgical reduction and fixation. These findings are valuable to polytrauma patients as the definitive surgery can be delayed to allow other more urgent, life-saving procedure, and physiological stabilization prior to surgical treatment of the spinopelvic dissociation. However, a temporary stabilization of the pelvic ring fracture with a pelvic external fixator should be in place while waiting for the definitive surgery. All three patients were free from Fat Embolism Syndrome although there was high incidence rate in closed fracture compared to open fracture reported by Bajuri et al. (21).

In the second patient, both longitudinal sacral fractures exit superiorly through L5/S1 facet joints, resulting in dislocation of both joints. In addition, the right transverse process of L5 spine was fractured too. The said fracture pattern above made it difficult to identify the insertion point for pedicle screws in L5 spine. We suggest caution in identifying L5 pedicles screw insertion point where there is instability between L5 and S1 due to facet fracture dislocation and L5 transverse process fracture as this may cause loss of bearing in the absence of normal anatomy.

The morbidly obese patient (BMI 50, class 3 morbid obesity) had a plain pelvic radiograph of the pelvis revealed subtle findings of sacral fracture. A CT scan was done based on clinical judgement and radiological uncertainty. In this patient, the lower back pain persisted despite maximal analgesia. Bhashyam et al. reported a higher wound complication rate with patients in class 3 morbid obesity following spinopelvic fixation (22). Fortunately, none of our patients develops wound complication such as infection or wound breakdown. The approach used in our surgery was midline incision with distal V-flap of the lumbosacral muscles to preserve blood supply. All three patients were discharged within a week and started rehabilitation earlier while in the ward.

## Conclusion

Our little experience with 3 patients found that spinopelvic fixation performed as late as 4 weeks is still a viable surgical treatment, particularly in a patient with multiple injuries (polytrauma) requiring further stabilization before undergoing major surgery such as spinopelvic fixation. We noticed minimal callus formation within the fracture site, making it mobile and feasible for reduction. The average waiting time was 3 weeks (ranging between 2 and 4 weeks).

All three patient had a similar construct which includes 4 polyaxial screw in L4 and L5 spine and 2 long polyaxial screws in the iliac bones, all connected with 5.5 mm stainless steel rod and a cross-link at the S2–S3 level. All construct supplemented with gullwing plate contoured using a 3.5 mm straight reconstruction plate. This construct allows for immediate mobilization without losing fracture reduction. No sacral decompression performed in all patients as none of the patients had a neurological deficit were reported pre and postoperatively. None of our patients developed surgical site wound infection.

Unstable multiplanar sacral fracture with spinopelvic dissociation treated with bilateral triangle osteosynthesis supplemented with gullwing plate is viable surgical treatment with an acceptable outcome, despite delayed up to 4 weeks.

## Data Availability

No datasets were generated or analyzed for this study.

## Author Contributions

MM, MB, AA, PG, MF, and AS conception and design of study, acquisition of data, analysis and interpretation of data, drafting manuscript, and approval of the version of the manuscript to be published.

### Conflict of Interest Statement

The authors declare that the research was conducted in the absence of any commercial or financial relationships that could be construed as a potential conflict of interest.
